# Interrelationships Between PD-L1, CTLA-4, IL-10 and IL-18 Expression in Oral Squamous Cell Carcinoma

**DOI:** 10.3390/cimb48080785

**Published:** 2026-08-01

**Authors:** Magdalena Miniuk, Joanna Reszeć-Giełażyn, Piotr Bortnik, Jan Borys, Kacper Koralewicz, Larys Lubowicki

**Affiliations:** 1 Department of Medical Pathomorphology, Medical University of Bialystok, 15-269 Bialystok, Poland; 2 Department of Maxillofacial and Plastic Surgery, Medical University of Bialystok, 15-276 Bialystok, Poland

**Keywords:** oral squamous cell carcinoma, OSCC, PD-L1, CTLA-4, interleukin 10 (IL-10), interleukin 18 (IL-18), immunotherapy, immune checkpoints

## Abstract

Oral squamous cell carcinoma (OSCC) is a malignancy characterized by a complex immunosuppressive tumor microenvironment. Immunomodulatory cytokines and immune checkpoints such as PD-L1 (programmed death-ligand 1) and CTLA-4 (cytotoxic T-lymphocyte-associated protein 4) play a crucial role in tumor development. Due to its high mortality rate, OSCC remains one of the major challenges in modern oncology. The aim of this retrospective study was to assess the expression of PD-L1, CTLA-4, IL-10, and IL-18 in paraffin-embedded tumor specimens and to analyze their associations with selected clinicopathological parameters in 79 patients treated surgically between 2011 and 2023. Most patients exhibited intermediate expression of PD-L1 (1–49%; 81.0%), CTLA-4 (1–49%; 87.3%), IL-18 (11–25%; 78.5%), and IL-10 (11–25%; 58.2%). PD-L1 and CTLA-4 expression showed a very strong association (*p* < 0.001, Cramer’s V = 0.823). IL-18 expression was negatively correlated with both PD-L1 (*p* = 0.017, Cramer’s V = 0.322) and CTLA-4 (*p* = 0.004, Cramer’s V = 0.322). A positive correlation between IL-18 and IL-10 expression was also observed (*p* < 0.001, Cramer’s V = 0.403). Among all analyzed biomarkers, only IL-10 expression showed a significant association with 5-year survival in univariate analysis (*p* = 0.009), with higher IL-10 expression associated with shorter survival. However, this finding did not retain statistical significance in multivariate analysis after adjustment for age and tumor stage. The results indicate a strong relationship between PD-L1 and CTLA-4, suggesting their complementary role in tumor-associated immunosuppression. The observed associations between IL-18 and PD-L1, and between IL-18 and CTLA-4, highlight the complexity of the mechanisms regulating the antitumor immune response. Although IL-10 did not retain independent prognostic significance, the findings from the univariate analysis may indicate a potential role for IL-10 in mechanisms related to tumor progression, warranting further investigation. Future studies should further clarify the interactions between cytokine-related immunoregulatory mechanisms and immune checkpoint pathways in OSCC, particularly in relation to IL-10, IL-18, PD-L1, and CTLA-4 expression.

## 1. Introduction

Oral squamous cell carcinoma (OSCC) is a malignant tumor arising from the mucosal epithelial cells of the oral cavity, pharynx, and larynx. It is characterized by aggressive behavior and can metastasize to regional cervical lymph nodes. Its global incidence has increased markedly in recent years, now exceeding 800,000 cases annually. Despite the availability of multiple therapeutic options, including surgery, chemotherapy, radiotherapy, and multimodal treatment strategies, overall survival rates have not significantly improved over the past three decades. Survival remains unsatisfactory at approximately 40–60% and may be as low as 19% in patients diagnosed at advanced stages [1,2,3,4].

Among patients with recurrent or metastatic OSCC, median overall survival generally remains below 12 months. The approval of immune checkpoint inhibitors, including nivolumab and pembrolizumab, by the U.S. Food and Drug Administration in 2016 marked a significant milestone in the treatment of these patients. Nivolumab, a monoclonal antibody targeting programmed cell death protein 1 (PD-1), was subsequently approved in Europe in 2017 and has been available in Poland since 2019. More than 400 patients have received nivolumab therapy during the past four years. Durable clinical responses have been observed in only 13–18% of patients, and the use of immune checkpoint inhibitors remains limited [5,6,7].

CTLA-4 (cytotoxic T-lymphocyte-associated protein 4) and PD-L1 (programmed death-ligand 1) play critical roles in regulating immune responses by suppressing T-cell activity. CTLA-4 inhibits T-cell proliferation during the priming phase, whereas PD-L1 expression on tumor cells and within the tumor microenvironment promotes T-cell exhaustion. PD-L1 may be expressed not only by cancer cells but also by tumor-infiltrating lymphocytes, macrophages, dendritic cells, fibroblasts, and endothelial cells. Overexpression of PD-L1 contributes to an immunosuppressive microenvironment and tumor progression [8].

In a recent multicenter observational study from Poland, nivolumab demonstrated clinical efficacy, low toxicity, and good tolerability in 324 patients with recurrent or metastatic head and neck squamous cell carcinoma (HNSCC) following progression on platinum-based therapy [7].

The efficacy of immunotherapy depends on complex interactions within the tumor microenvironment, including cytokines that modulate immune responses and exert context-dependent effects on tumor development. Interleukin-10 (IL-10), a pleiotropic cytokine, exerts immunosuppressive effects mediated by regulatory T cells (Tregs), myeloid-derived suppressor cells, and tumor-associated macrophages. Under certain conditions, IL-10 may also enhance antitumor immunity: for example, through activation of CD8+ T lymphocytes [9].

In a study by Emmerich et al., IL-10 induced the activation and expansion of tumor-resident CD8+ T cells, resulting in effective tumor control in animal models [10].

Regulatory T cells (Tregs) are among the key mediators of immunosuppression in OSCC. By secreting IL-10, TGF-β, and IL-35 and expressing CTLA-4, they suppress effector immune responses. This immunosuppressive cascade inhibits CD8+ T-cell activation, attenuates IL-2-dependent signaling, impairs NK-cell function, and disrupts dendritic cell activation, ultimately promoting tumor immune evasion and disease progression [11]. Interleukin-18 (IL-18), an immunomodulatory cytokine that induces IFN-γ production, may be produced directly by HNSCC cells [12]. Elevated serum IL-18 levels are frequently observed in patients with HNSCC, suggesting its potential role as a disease biomarker [13,14].

Interferon-γ has been detected at the invasive margins of PD-L1-positive tumors and in regions infiltrated by tumor-infiltrating lymphocytes (TILs), whereas it is absent in PD-L1-negative tumors. These findings suggest that activated T cells may initiate a negative feedback mechanism by secreting cytokines that induce PD-L1 expression, thereby promoting adaptive immune resistance and enabling tumor immune evasion [15]. Immune checkpoint inhibitor (ICI) therapy targeting the PD-1/PD-L1 and CTLA-4 pathways restores antitumor immune responses by blocking inhibitory signaling pathways in T lymphocytes. Despite the proven clinical efficacy, only a subset of patients achieves durable clinical benefit. This may be attributable to the highly immunosuppressive tumor microenvironment in OSCC, characterized by impaired T-cell activation, induction of immune tolerance, and multiple mechanisms of tumor immune evasion [16].

To improve the understanding of OSCC biology and identify potential prognostic biomarkers, it is important to investigate the relationships between immune checkpoint molecules, cytokines, and clinicopathological parameters.

## 2. Materials and Methods

### 2.1. Patients and Samples

The cohort consisted of 79 patients diagnosed with oral squamous cell carcinoma who underwent primary surgical treatment between 2011 and 2023 at the Department of Maxillofacial Surgery, University Clinical Hospital, Białystok, Poland. All patients underwent surgical tumor resection and neck dissection, followed by adjuvant radiotherapy or chemotherapy when clinically indicated. The study was conducted with the approval of the Bioethics Committee of the Medical University of Białystok (decision no. APK.002.249.2024). All tumor specimens included in the analysis were derived from archival diagnostic material collected during routine surgical treatment. Informed consent was obtained from all individual participants included in the study.

### 2.2. Methodology

Tissue specimens collected during surgery were fixed in 4% paraformaldehyde for 24 h. Subsequently, the specimens were dehydrated and embedded in paraffin using the Excelsior AS tissue processor (Thermo Fisher Scientific). The resulting tissue blocks were sectioned at 3 μm and mounted on microscope slides (Thermo Fisher). To visualize and assess the tissue architecture, hematoxylin and eosin staining was performed using dedicated Tissue-Tek^®^ H&E reagents (Sakura Finetek) for the Tissue-Tek Prisma^®^ Plus automated staining system, according to the manufacturer’s protocol.

### 2.3. Immunohistochemical Reaction (IHC)

Paraffin sections (3 μm thick) were dried in an incubator at 56 °C for one hour, deparaffinized in xylene, rehydrated through a graded series of ethanol solutions, rinsed in distilled water, and treated with 3% hydrogen peroxide to block endogenous peroxidase activity. Antigen retrieval was performed in a PT Link system (Agilent Technologies) using Target Retrieval Solution (TRS, pH 6.0 or 9.0) at 97 °C for 20 min. After cooling, the slides were incubated with blocking serum to reduce nonspecific background staining, followed by overnight incubation at 4 °C in a humidified chamber with appropriately prepared primary antibodies. Antibodies, antigen retrieval conditions, and dilution factors are listed in Table 1. CTLA-4, IL-18, and IL-10 were visualized using the EnVision+ Dual Link System-HRP (DAB+) detection system (Agilent Technologies), which is compatible with mouse and rabbit primary antibodies. PD-L1 staining was performed according to the manufacturer’s protocol for the PD-L1 IHC 22C3 pharmDx kit (Code SK006; Agilent Technologies) using the Autostainer Link 48 platform. After completion of the immunohistochemical procedure, nuclear counterstaining was performed with Mayer’s hematoxylin, followed by dehydration in graded alcohol and xylene solutions. The stained slides were coverslipped using the Tissue-Tek Film automated coverslipper (Tissue-Tek^®^ Film^®^, Cat. No. 4770-E; Sakura Finetek) and subsequently scanned using the PANNORAMIC 250 Flash III DX scanner (3DHISTECH).

To improve reproducibility, all immunohistochemical assessments of representative tumor areas were performed independently by two experienced pathologists blinded to the clinical data. Both used the same predefined scoring criteria. In cases of equivocal or borderline staining intensity, the slides were jointly re-evaluated to achieve consensus. To account for potential intratumoral heterogeneity, biomarker expression was determined by assessing at least 100 viable cells in representative hotspots with the highest density of immuno-positive tumor cells. For this purpose, predefined scoring criteria were used. PD-L1 expression was assessed using the Tumor Proportion Score (TPS), which represents the percentage of viable tumor cells showing membranous staining relative to all viable tumor cells in the specimen.

PD-L1 expression was scored as follows:

Number of tumor cells evaluated: ≥100 cells.

Tumor Proportion Score (TPS):

TPS < 1%: negative staining (0).

TPS 1–49%: intermediate expression (1).

TPS ≥ 50%: strong expression (2).

CTLA-4 expression was evaluated semi-quantitatively based on the percentage of positively stained tumor cells using the same percentage cut-off values as those applied for PD-L1 to facilitate comparison between the analyzed biomarkers [17,18].

CTLA-4 expression was scored as follows:

Number of tumor cells evaluated: ≥100 cells.

<1% positive tumor cells: negative staining (0).

1–49% positive tumor cells: intermediate expression (1).

≥50% positive tumor cells: strong expression (2).

Validation of the PD-L1 clone 22C3 antibody was based on its widespread use as a companion diagnostic for pembrolizumab. Negative controls were included for all analyzed markers (PD-L1, CTLA-4, IL-10, and IL-18). Normal tonsil tissue was used as a control tissue.

The scoring system for IL-10 and IL-18 was based on the literature [19,20].

Expression was assessed as follows:

0: no positive cells or <10% positive cells.

1: 11–25% positive cells.

2: 26–50% positive cells.

3: >50–100% positive cells.

### 2.4. Statistical Methods

Statistical analysis were performed using IBM SPSS 26.0 Statistics software. Descriptive statistics appropriate for the level of measurement were applied. For qualitative variables, frequencies and percentages were reported. For quantitative variables, measures such as the mean, median, and standard deviation were used.

The normality of distribution was assessed using the Kolmogorov–Smirnov test. Depending on the nature of the data, relationships between variables were analyzed using Spearman’s rank correlation coefficient (rho), the Mann–Whitney U test, and the Kruskal–Wallis test. Associations between categorical variables were evaluated using Pearson’s chi-square test, and their strength was measured with Cramér’s V coefficient.

Multiple regression analysis was used to assess the effects of age and BMI on PD-L1 expression. Receiver operating characteristic (ROC) analysis was performed to determine the optimal PD-L1 cut-off value for predicting 5-year survival. To identify independent factors associated with 5-year survival, multivariate logistic regression analysis was performed. A *p*-value < 0.05 was considered statistically significant.

## 3. Results

The analyzed material included incisional biopsy specimens of primary tumors (histopathologically confirmed oral squamous cell carcinomas), recurrent lesions, and tissue specimens obtained from regional cervical lymph nodes. Cases were categorized according to TNM classification, clinical stage, and histological grade (G) based on the eighth edition of the American Joint Committee on Cancer (AJCC). Additionally, data regarding sex, age, tumor location, 5-year survival status, tobacco and alcohol use, body mass index (BMI), the occurrence of local recurrence, nodal recurrence, and distant metastases were analyzed. The characteristics of the study cohort are presented in Table 2.

The study cohort was predominantly composed of male patients (67.1%), with a mean age of 61.5 years. Most tumors originated from the tongue or floor of the oral cavity. Stage IV disease constituted the largest subgroup of patients. Histopathologically, the majority of cases represented moderately differentiated carcinomas (G2).

**Figure 1:**
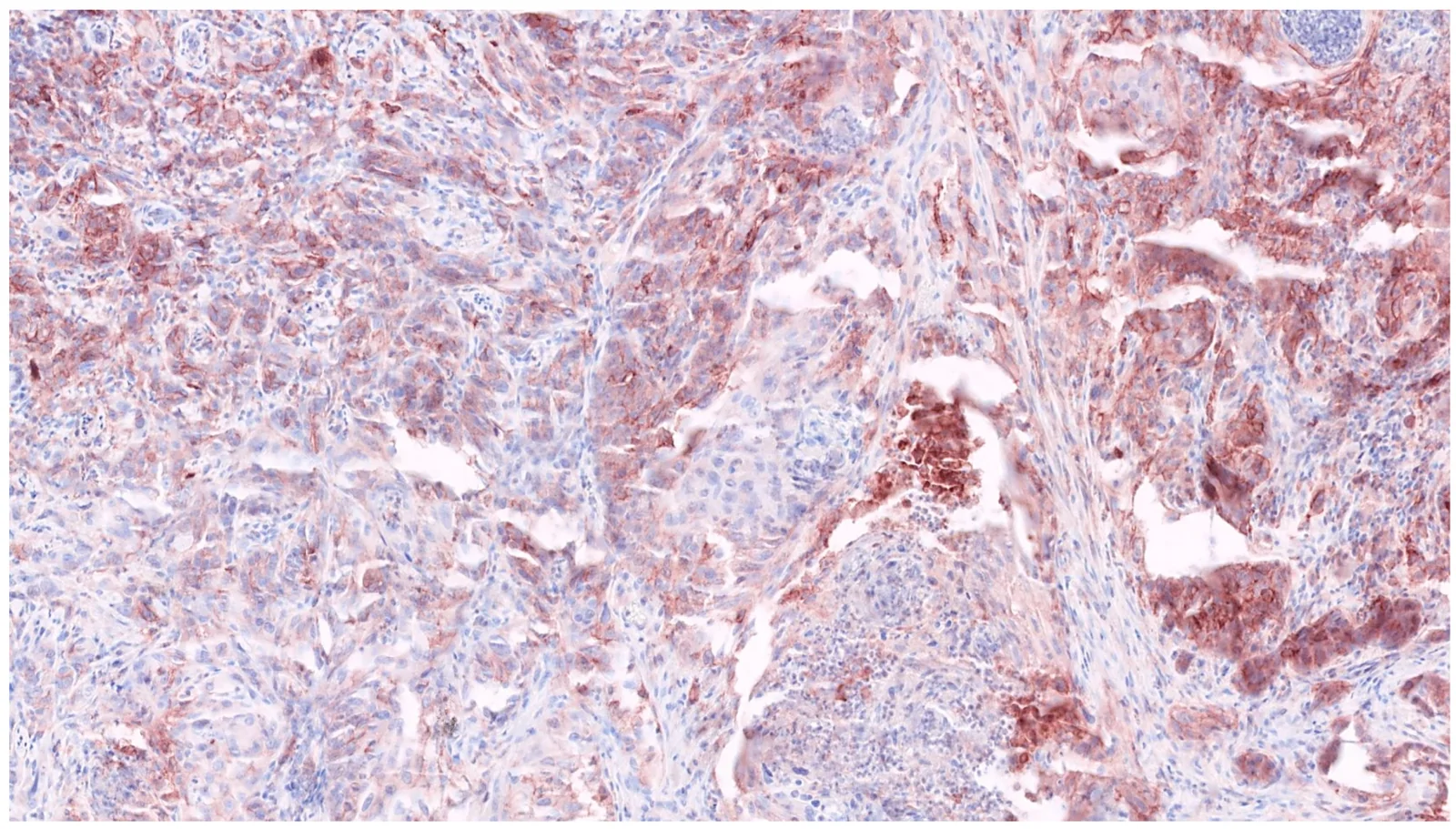
Intermediate CTLA-4 expression in squamous cell carcinoma cells of the floor of the oral cavity and tongue. Magn. ×200.

**Figure 2:**
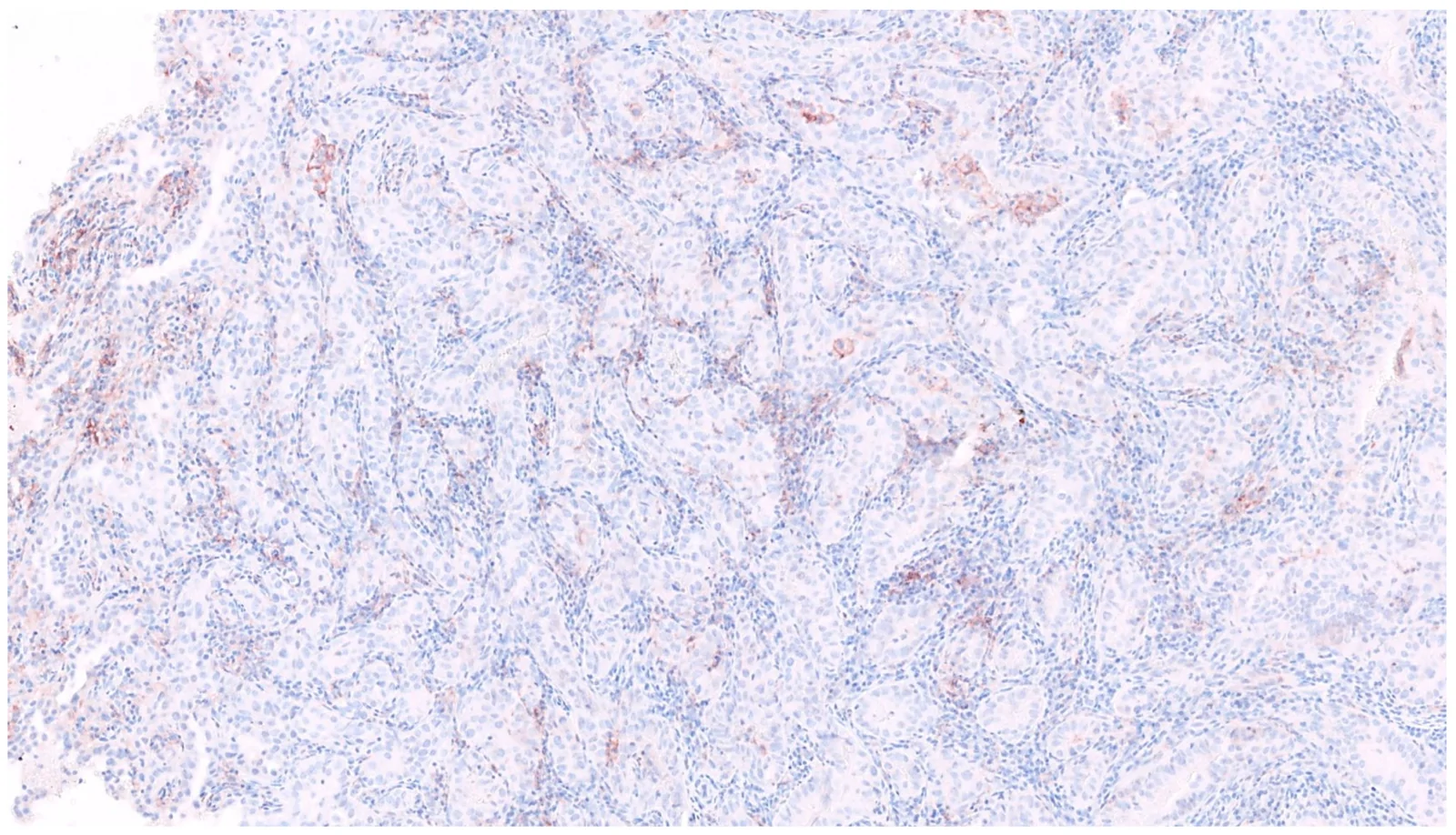
Intermediate PD-L1 expression in squamous cell carcinoma cells of the floor of the oral cavity and tongue. Magn. ×100.

**Figure 3:**
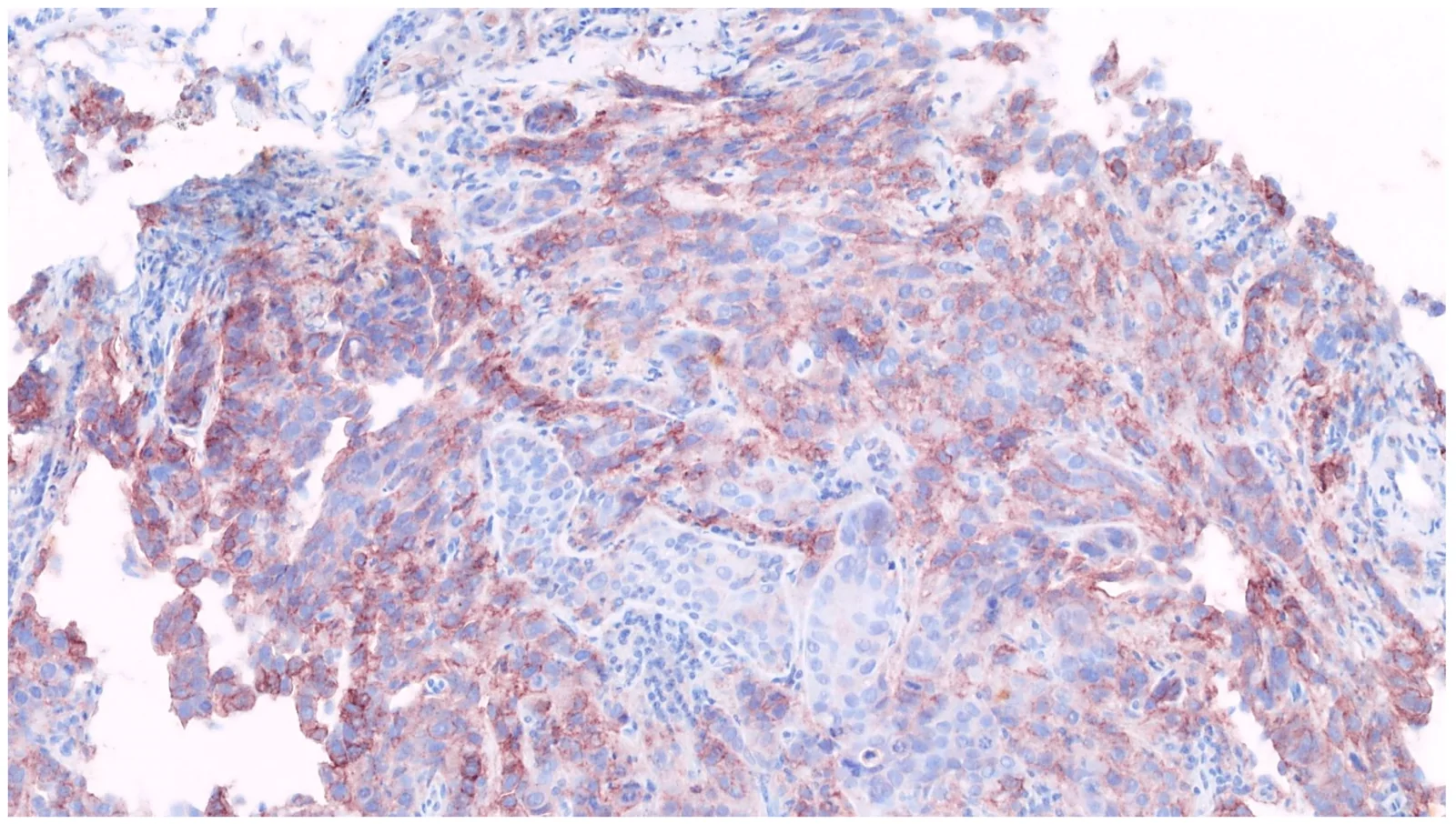
Strong PD-L1 expression in squamous cell carcinoma cells of the floor of the oral cavity and tongue. Magn. ×200.

**Figure 4:**
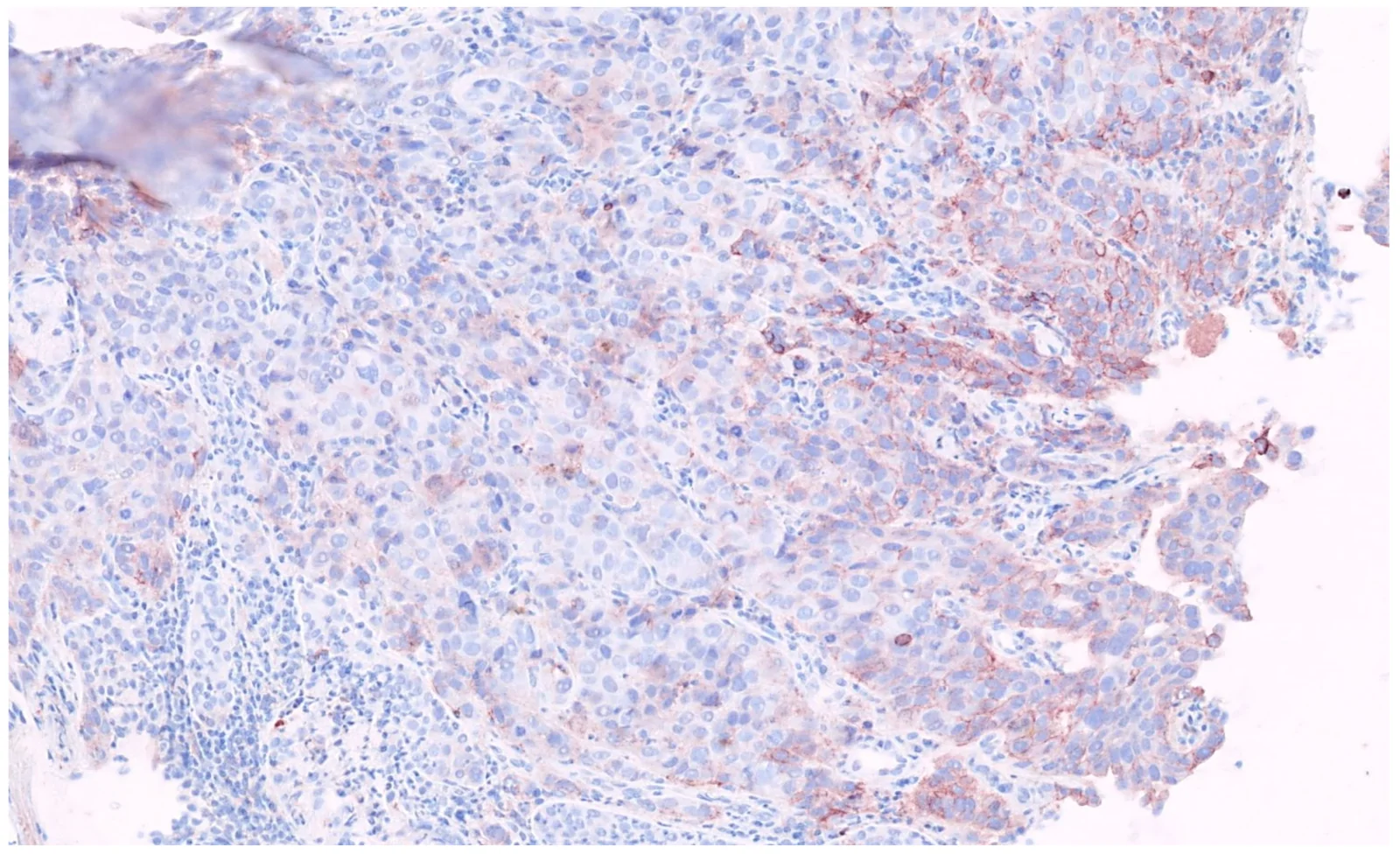
Intermediate IL-10 expression in squamous cell carcinoma cells of the floor of the oral cavity and tongue. Magn. ×200.

**Figure 5:**
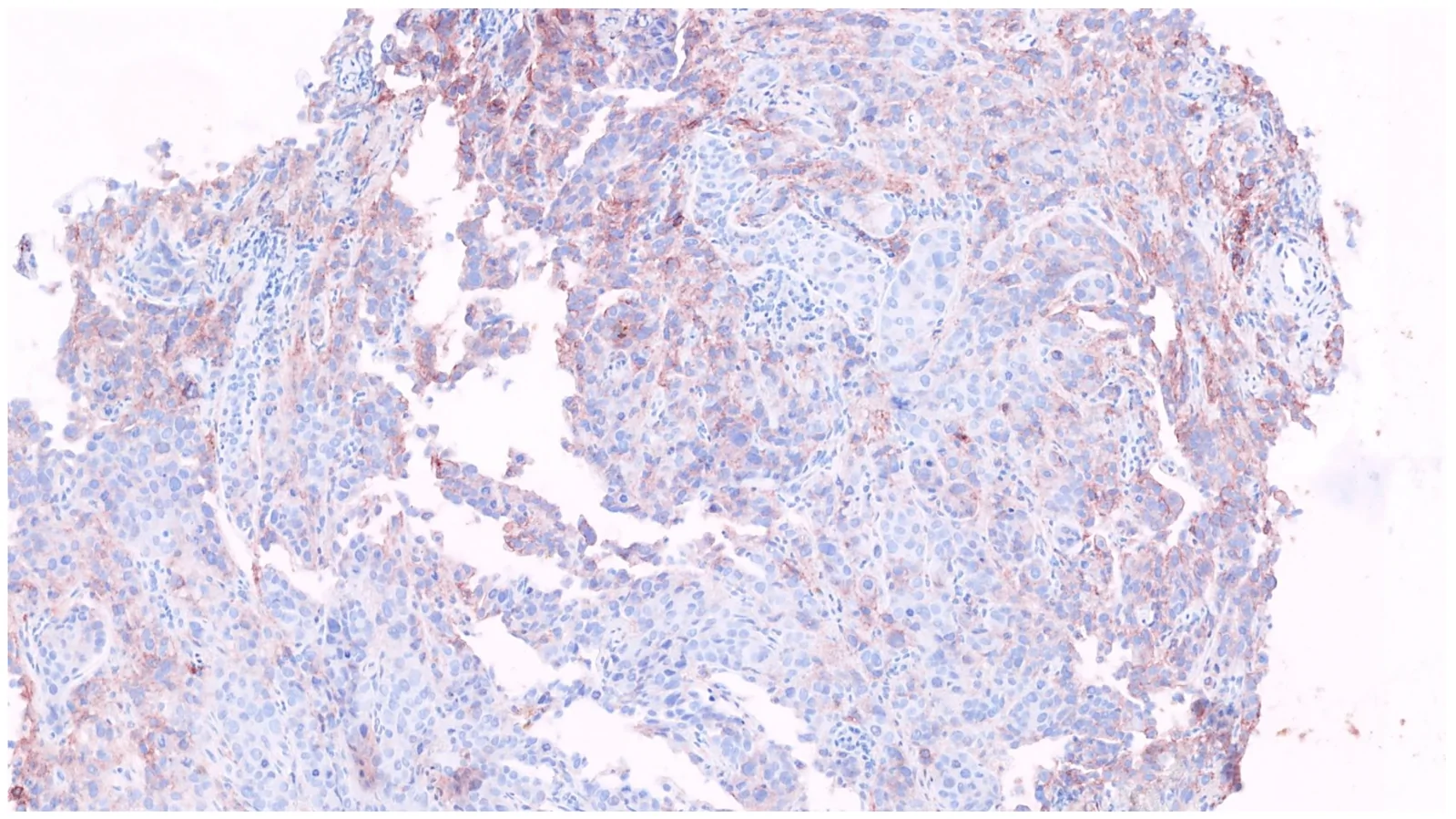
Intermediate IL-18 expression in squamous cell carcinoma cells of the floor of the oral cavity and tongue. Magn. ×200.

PD-L1 and CTLA-4 expression most commonly ranged between 1–49%. lub Intermediate CTLA-4 expression in squamous cell carcinoma cells of the oral floor and tongue is shown in Figure 1. The mean proportion of PD-L1-positive cells was 27.03 ± 17.48%. PD-L1 showed considerable variability and a non-normal distribution. Representative images of intermediate and strong PD-L1 expression in squamous cell carcinoma cells of the floor of the oral cavity and tongue are shown in Figure 2 and Figure 3, respectively. Similarly, IL-18 and IL-10 were predominantly expressed at intermediate levels, with fewer patients showing low expression (≤10% positive cells) or high expression (26–50% positive cells). Intermediate IL-10 and IL-18 expression in squamous cell carcinoma cells of the floor of the oral cavity and tongue is illustrated in Figure 4 and Figure 5, respectively. Notably, no patients exhibited strong CTLA-4 expression (50–100% positive cells) or very high IL-18 and IL-10 expression (>50% positive cells). Detailed results are presented in Table 3.

**Figure 6:**
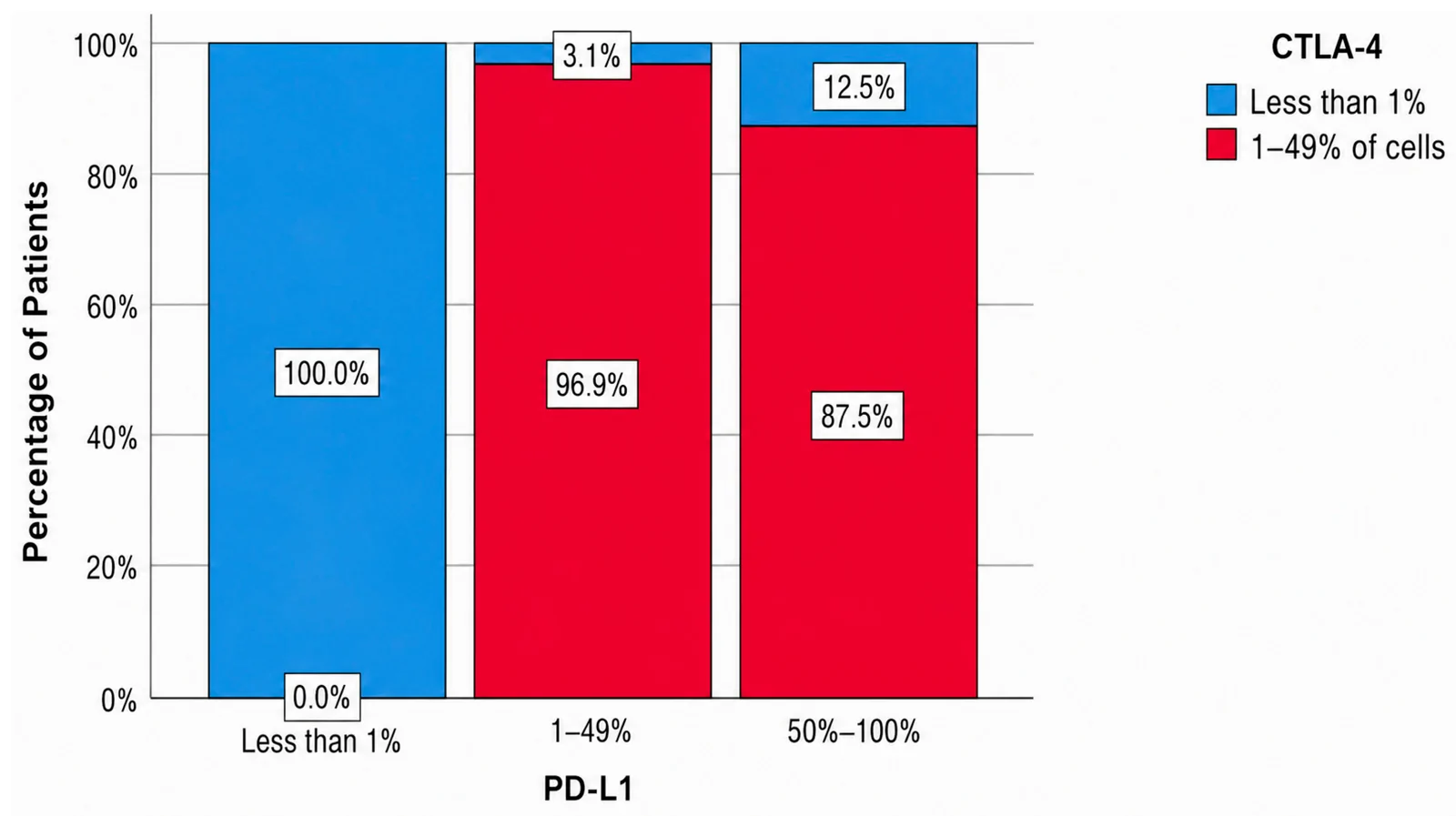
PD-L1 and CTLA-4 expression. Figure 6 shows the percentage distribution of CTLA-4 expression according to PD-L1 expression levels. CTLA-4 negativity was observed predominantly in PD-L1-negative tumors, whereas intermediate CTLA-4 expression (1–49% of cells) predominated among patients with PD-L1-positive tumors, including both the intermediate (1–49%) and high (50–100%) PD-L1 expression groups.

**Figure 7:**
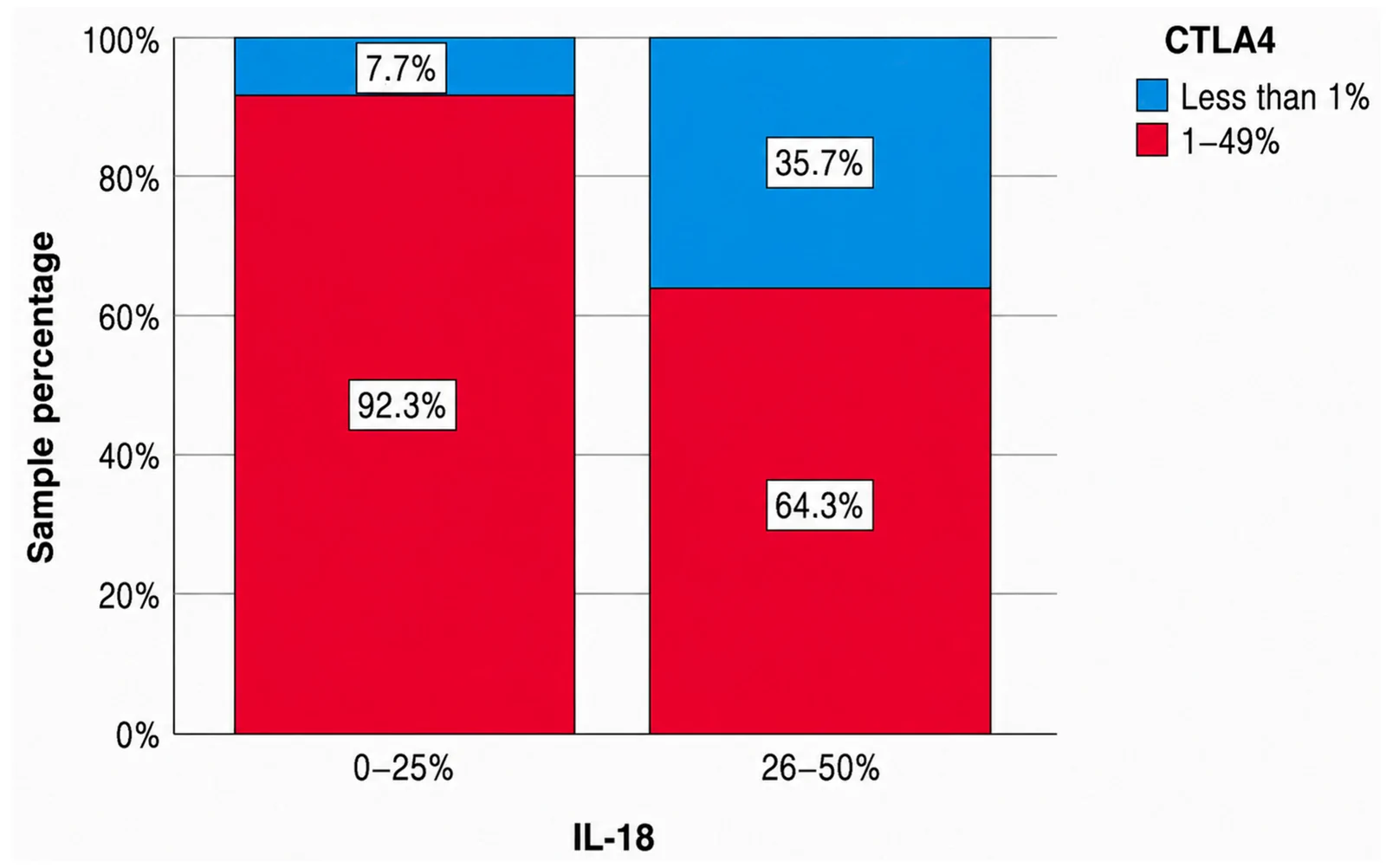
IL-18 and CTLA-4. Figure 7 shows the percentage distribution of CTLA-4 expression according to IL-18 expression levels.

**Figure 8:**
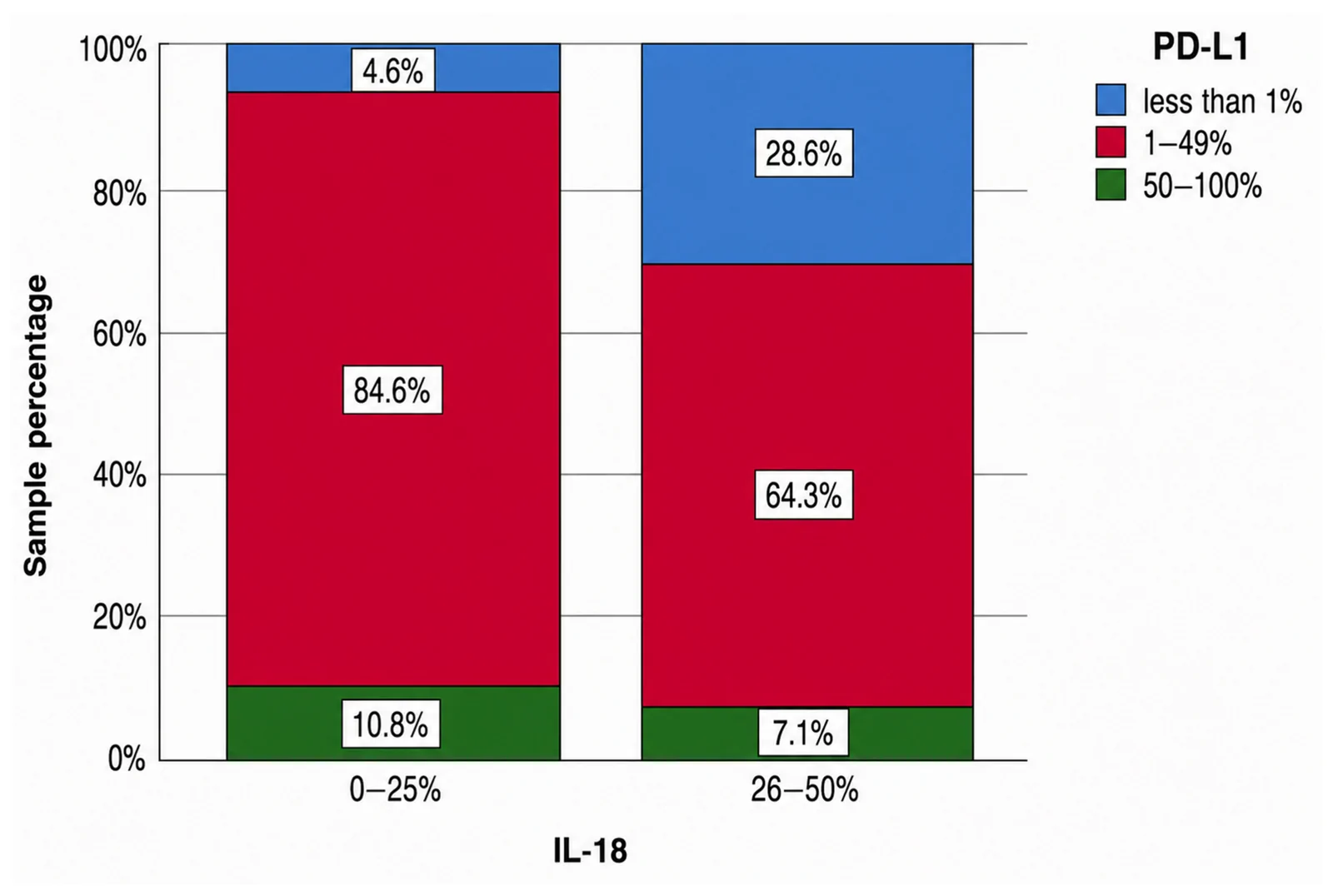
IL-18 and PD-L1. Figure 8 shows the percentage distribution of PD-L1 expression according to IL-18 expression levels.

**Figure 9:**
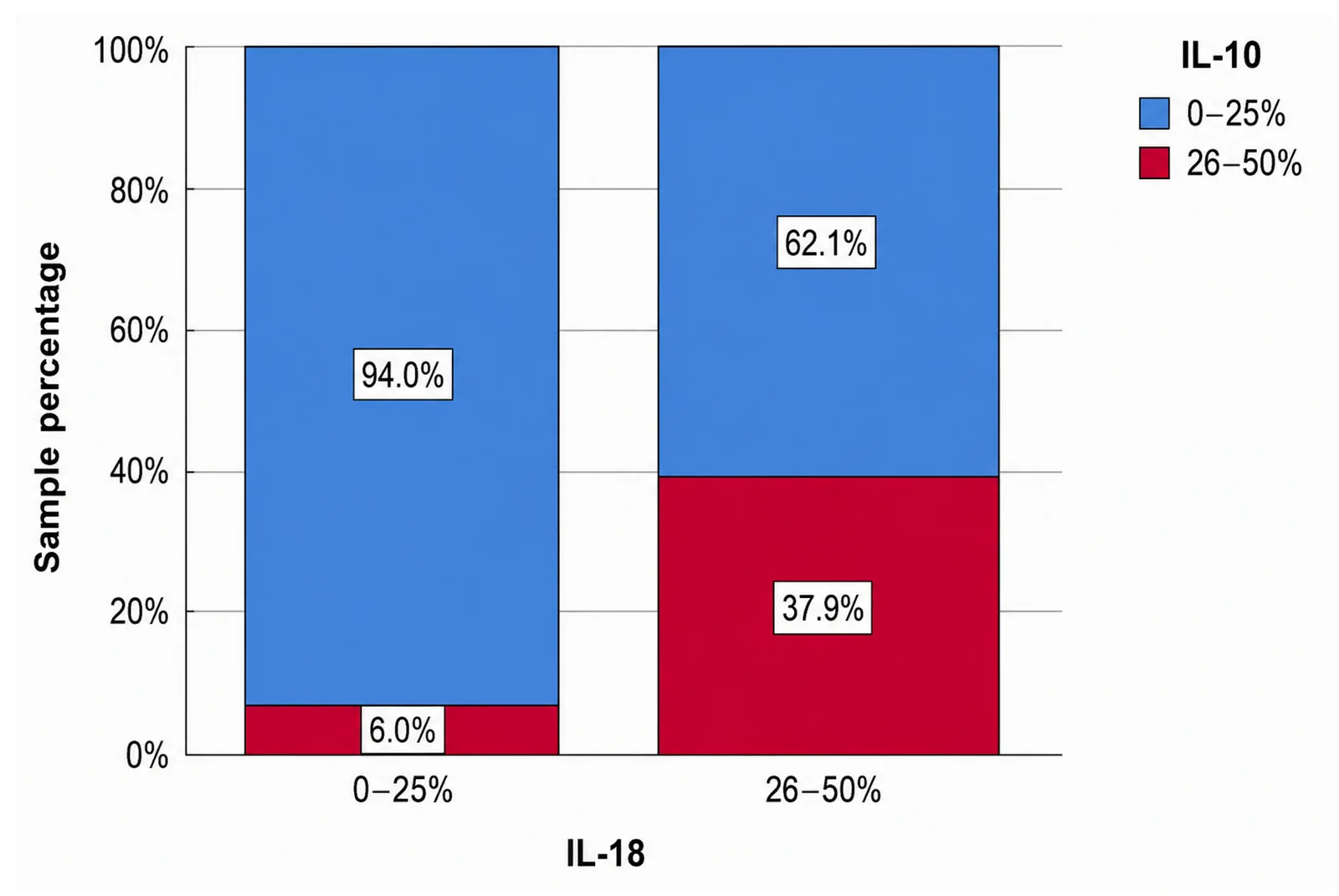
IL-10 and IL-18. Figure 9 shows the percentage distribution of IL-10 expression according to IL-18 expression levels.

A very strong association was observed between PD-L1 and CTLA-4 expression (*p* < 0.001, Cramér’s V = 0.823). The absence of PD-L1 expression was associated with the absence of CTLA-4 expression, whereas PD-L1-positive tumors (both intermediate, 1–49%, and high, 50–100%) were associated with intermediate CTLA-4 expression (1–49%) (Table 4, Figure 6). Moderate correlations were also found between IL-18 and both CTLA-4 (Table 5, Figure 7) and PD-L1 (Table 6, Figure 8). No significant associations were identified between IL-10 and CTLA-4 (Table 7a) or PD-L1 (Table 7b). In contrast, IL-10 showed a moderate positive correlation with IL-18. Due to the low number of patients with IL-10 and IL-18 expression ≤10% (score 0), scores 0 and 1 were combined for statistical analyses. Consequently, the categories ≤10% positive cells and 11–25% positive cells were analyzed as a single group (0–25%), allowing for more robust statistical comparisons and reducing the impact of sparse subgroup counts (Table 8, Figure 9).

**Figure 10:**
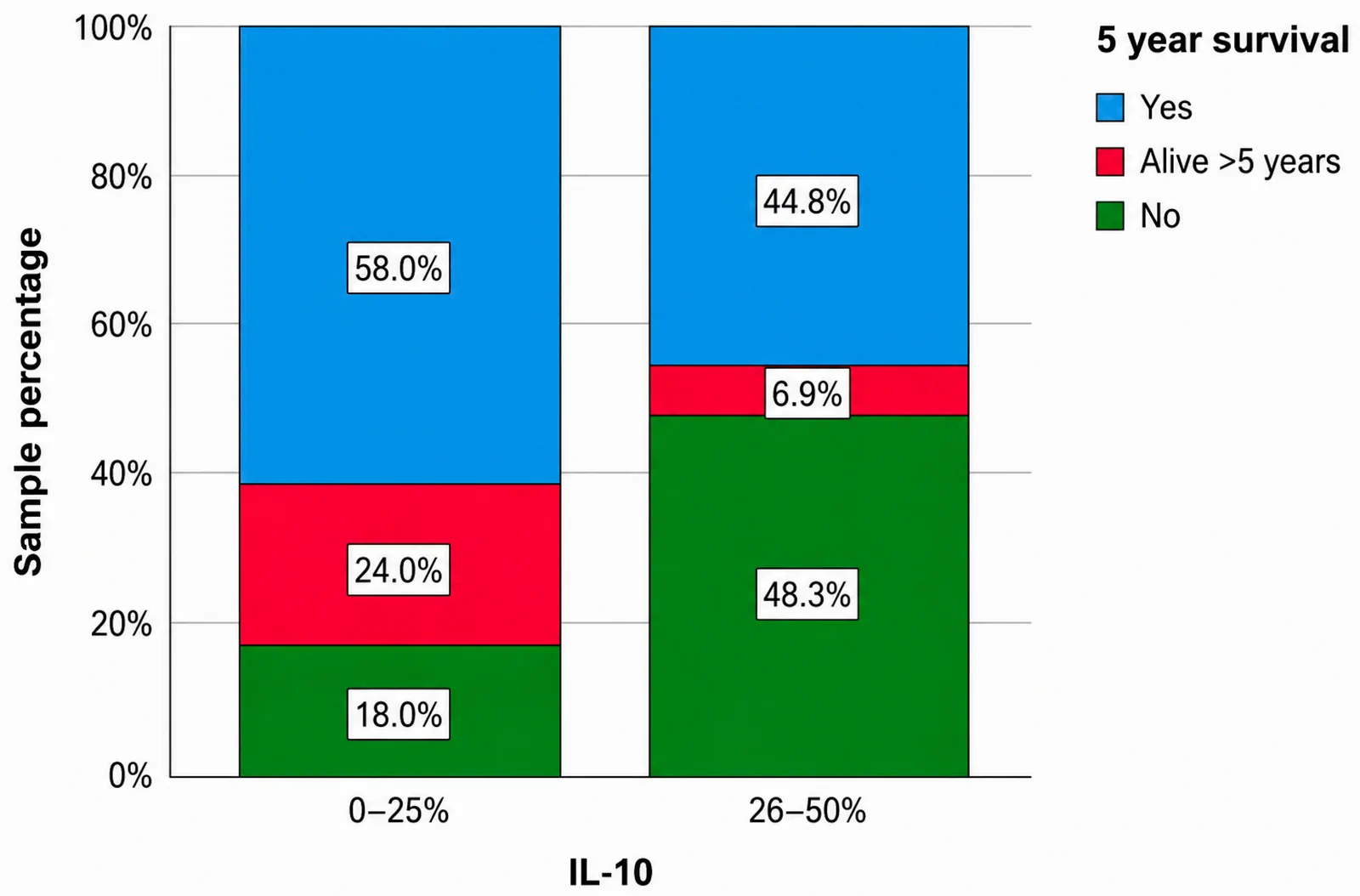
IL-10 and 5-year survival. Figure 10 shows the percentage distribution of 5-year survival according to IL-10 expression levels.

Only IL-10 expression showed a statistically significant association with 5-year survival in univariate analysis (*p* = 0.009, Cramér’s V = 0.290). Patients with higher IL-10 expression were less likely to achieve 5-year survival. Lower IL-10 expression was associated with better survival outcomes (Table 9, Figure 10). In multivariate logistic regression analysis adjusted for age and tumor stage, IL-10 expression did not remain an independent prognostic factor. Tumor stage was the only independent prognostic factor associated with 5-year survival (Table 10). A non-significant trend toward poorer 5-year survival was observed in older patients.

No significant differences in BMI and age were observed across PD-L1, CTLA-4, IL-18, and IL-10 expression levels. However, a slight, non-significant tendency toward higher BMI was noted in patients with lower CTLA-4 and higher IL-10 expression (Table S5 “Supplementary Materials”). Additionally, no significant differences in PD-L1, CTLA-4, IL-10, or IL-18 expression were observed according to the primary tumor location within the oral cavity (Tables S1–S4 “Supplementary Materials”). No significant associations were found between the expression of the analyzed biomarkers and the remaining clinicopathological factors, including sex, clinical stage, T and N category, smoking status, alcohol consumption, distant metastases, or local recurrence (Tables S1–S4 “Supplementary Materials”). Similarly, no significant associations were found between PD-L1, CTLA-4, or IL-18 expression and 5-year survival (Tables S1–S4 “Supplementary Materials”).

## 4. Discussion

The immune response in OSCC is regulated by a complex network of activating and inhibitory signals within the tumor microenvironment. While the PD-1/PD-L1 and CTLA-4 pathways are among the best-characterized immune checkpoints, they constitute only part of the broader immunoregulatory network involved in antitumor immunity. Cytokines such as IL-10 and IL-18 modulate antitumor immune responses and can influence mechanisms of tumor evasion depending on the local context. Given the variable clinical responses to checkpoint inhibitors, a better understanding of the interactions among multiple immunosuppressive pathways is essential for improving therapeutic outcomes.

In the studied cohort (*n* = 79), intermediate PD-L1 expression (1–49%; class 1) was the predominant staining pattern, observed in 81% of patients. In contrast, high PD-L1 (≥50%; class 2) and negativity (<1%; class 0) were markedly less common. The observed distribution of PD-L1 status is consistent with previous reports across different PD-L1 assessment methodologies [16,21,22].

PD-L1 expression in OSCC exhibits considerable variability, which may be influenced by both technical and biological factors. Technical factors include differences in antibody selection, scoring methodologies, and cut-off values, whereas biological include interpatient variability, tumor heterogeneity, disease stage, and temporal changes during tumor progression. Therefore, PD-L1 expression data should be interpreted with caution. Evidence suggests that PD-L1 expression levels may vary according to the timing of assessment (at diagnosis, recurrence, or disease progression) and between primary and secondary lesions [23,24].

For CTLA-4, intermediate expression (1–49%; class 1) was the predominant pattern, observed in 87.3% of patients. CTLA-4 negativity (<1%; class 0) was observed in 12.7% of patients, whereas high CTLA-4 (≥50%; class 2) was not detected in the studied cohort. These findings are consistent with the limited body of literature available on CTLA-4 distribution in OSCC [17,25].

In our study, we observed a strong association between PD-L1 and CTLA-4 expression. The absence of PD-L1 was associated with the absence of CTLA-4, whereas the presence of PD-L1 (both intermediate, 1–49%, and high, ≥50%) was associated with intermediate CTLA-4 levels (1–49%) (*p* < 0.001, Cramér’s V = 0.823). These findings suggest that both immune checkpoints may contribute to a common immunosuppressive profile within the tumor microenvironment. A previous study [26] demonstrated an association between CTLA-4 and PD-1 (the receptor for PD-L1) expression on regulatory T cells (Tregs), which may reflect similar mechanisms operating within the immunosuppressive tumor microenvironment. The co-occurrence of CTLA-4 and PD-L1 in OSCC highlights the potential value of therapeutic strategies targeting multiple immune checkpoints simultaneously. To the best of our knowledge, no previous studies have reported such a strong association between CTLA-4 and PD-L1 in OSCC, although there is evidence suggesting overlapping immunoregulatory mechanisms involving CTLA-4 on T cells and PD-L1 on tumor cells [19]. These findings may provide new insights into the co-occurrence of CTLA-4 and PD-L1 within the immunosuppressive tumor microenvironment of OSCC and support further investigation of combined therapeutic strategies targeting multiple immune checkpoints. We also identified inverse associations of IL-18 expression with PD-L1 (*p* = 0.017, Cramér’s V = 0.322) and CTLA-4 (*p* = 0.004, Cramér’s V = 0.322). In contrast, IL-10 showed a positive association with IL-18 (*p* < 0.001, Cramér’s V = 0.403). Lower PD-L1 and CTLA-4 levels were accompanied by higher IL-18, while elevated IL-18 correlated with increased IL-10 levels. These findings are consistent with previous reports highlighting the complex role of immune signaling pathways in tumor progression [27].

PD-L1 expression may be induced by cytokines such as IFN-γ, produced by tumor-infiltrating lymphocytes. This represents a mechanism of adaptive immune resistance [28]. Given that IL-18 can stimulate IFN-γ production, one might expect a positive association between IL-18 and PD-L1; however, this was not observed in our study. The observed discrepancy may reflect the dual and context-dependent role of IL-18 within the OSCC tumor microenvironment. Chronic cytokine stimulation may promote both activation of antitumor immunity and immune dysregulation. A positive association between IL-18 and PD-1 expression on tumor-infiltrating NK cells has been reported by Liou et al., where it was linked to functional exhaustion and poorer prognosis in nasopharyngeal carcinoma [29]. Notably, these findings were specific to immune rather than tumor cells. In contrast, our results demonstrated inverse associations between IL-18 and both PD-L1 and CTLA-4 expression in OSCC tumor cells. Several studies have shown that IL-18 may promote the accumulation of myeloid-derived suppressor cells (MDSCs), and that its blockade can enhance the efficacy of anti-PD-1 therapy. This suggests that IL-18 modulation could potentially improve the effectiveness of PD-1/PD-L1-targeted immunotherapy [30]. IL-18 plays an important role in activating antitumor immune responses by stimulating IFN-γ production and enhancing the activity of NK cells and T lymphocytes. The BPT567 conjugate, combining IL-18 activity with PD-1 blockade, demonstrated greater antitumor efficacy than anti-PD-1 monotherapy. It amplified NK- and CD8+ T-cell activity, increased IFN-γ production, and ultimately promoted more effective tumor elimination [31].

These findings also suggest that modulation of the IL-18 pathway may represent a promising strategy to potentiate immunotherapy, particularly when combined with immune checkpoint inhibitors.

We did not observe significant correlations between IL-10 and PD-L1 or CTLA-4 levels. This contrasts with reports suggesting positive associations between inflammatory cytokines, including IL-10, and PD-L1 expression [8]. The positive correlation between IL-10 and IL-18, together with the inverse correlations of IL-18 with PD-L1 and CTLA-4, suggests the coexistence of both pro-inflammatory and immunosuppressive mechanisms within the tumor microenvironment, reflecting the complexity of its regulatory network.

IL-10, as a key immunosuppressive cytokine, modulates antitumor immune responses and promotes the differentiation of immunosuppressive cell populations, facilitating tumor immune evasion [5]. Importantly, IL-10 is involved in regulating and resolving inflammation. It influences the activity and differentiation of multiple immune cells, including T, B, and NK cells [32]. Thus, higher IL-10 levels and an immunosuppressive tumor microenvironment correlate with a poorer prognosis [33].

A positive correlation between IL-18 and IL-10 has been described in breast cancer, where both cytokines were associated with lymph node metastasis, suggesting their contribution to tumor progression [34]. In our study, no association with nodal metastases was observed. However, we noted a non-significant trend toward more frequent local recurrence in patients with higher IL-18 levels. Similar findings linking IL-18 and IL-10 with recurrence have been reported in colorectal cancer [35].

Cytokines can recruit immune cells to the tumor microenvironment and induce the expression of immune checkpoint molecules, such as PD-1 and TIM-3, thereby promoting tumor immune evasion. IL-10 contributes to the maintenance of an immunosuppressive tumor microenvironment by promoting the activity of tumor-associated macrophages (TAMs) and the expansion of regulatory T cells (Tregs), while suppressing the activity of effector CD8+ T cells [11]. In this context, the positive correlation between IL-10 and IL-18 observed in our study may reflect the coexistence of pro-inflammatory responses and immunoregulatory mechanisms that limit excessive immune activation. Interestingly, combined modulation of the IL-10 pathway and immune checkpoint blockade has been shown to enhance antitumor immune responses by increasing CD8+ T-cell activity and inhibiting tumor growth [36].

Our findings demonstrate a significant correlation (*p* = 0.009) between IL-10 expression and 5-year survival in univariate analysis. Patients with lower IL-10 expression (classes 0 and 1) exhibited a markedly higher 5-year survival rate than those with intermediate IL-10 expression (class 2). However, in multivariate analysis, this association did not retain statistical significance after adjustment for age and tumor stage. Thus, IL-10 cannot be considered an independent prognostic factor for 5-year survival. The univariate analysis suggests that IL-10 may be involved in tumor progression and immune regulation, although its prognostic impact may depend on other clinicopathological factors and requires further investigation.

Studies on nasopharyngeal carcinoma and non-Hodgkin lymphoma have reported an association between high IL-10 expression and poorer prognosis, including reduced survival, with IL-10 recognized as an independent prognostic factor in these malignancies [37,38]. Meta-analyses have linked elevated IL-10 levels not only with unfavorable prognosis, but also with shorter progression-free survival and more advanced disease. Increased IL-10 expression has also been observed in OSCC compared with normal tissues. These findings support its potential involvement in mechanisms of tumor immune escape [36].

The therapeutic potential of IL-10 pathway modulation has attracted increasing interest. In experimental models, the use of an IL-10 fusion protein in combination with immune checkpoint inhibitors enhanced antitumor efficacy while maintaining a favorable safety profile [39]. This effect was associated with increased CD8+ T-cell activity and IFN-γ production. Inhibition of IL-10 signaling may partially restore the cytotoxic function of effector cells against tumor cells [36].

We found no significant associations between the analyzed clinicopathological factors and the levels of CTLA-4, PD-L1, IL-18, or IL-10.

It is important to note that the analyzed material spanned a relatively long period (2011–2023), which may have affected the observed protein expression levels. Prolonged storage of paraffin-embedded OSCC tissue blocks may result in reduced PD-L1 levels. This applies to both TPS and CPS scoring systems, which, after several years of storage, may differ substantially from results obtained from freshly stained material. These observations suggest that the archiving period should be considered a potential source of bias in retrospective studies, as long-term storage may result in antigen degradation and underestimation of PD-L1 expression [40,41]. Although this phenomenon cannot be excluded in our study, no apparent association was observed between the year of tissue collection and PD-L1 levels, since both high and absent PD-L1 expression occurred in specimens obtained throughout the study period.

In addition to the extended study timeframe, several other limitations should be acknowledged. These include the retrospective single-center design and the lack of precise survival data for all patients. Consequently, survival analyses were limited to a 5-year follow-up period. Furthermore, the relatively small sample size, particularly within certain clinicopathological subgroups, may have reduced the statistical power to detect weaker associations. Therefore, non-significant findings should be interpreted with caution, as they may partly reflect a type II error. Another potential limitation of the present study is the intratumoral heterogeneity of biomarker expression. Although the assessment was performed in representative hot-spot areas, regional differences in marker expression within the tumor may have influenced the semiquantitative evaluation. Additional limitations include the lack of quantitative TIL assessment and the absence of data on the Worst Pattern of Invasion (WPOI), tumor budding, surgical margin status, perineural invasion (PNI), lymphovascular invasion (LVI), extranodal extension (ENE), and HPV/p16 status. However, the study cohort consisted exclusively of patients with oral squamous cell carcinoma, in which HPV status is not routinely incorporated into the AJCC 8th edition staging system and has a less well-established prognostic role than in oropharyngeal squamous cell carcinoma [42].

An increasing body of evidence suggests that HPV may influence the PD-1/PD-L1 axis and the composition of the tumor immune microenvironment. HPV-positive tumors are often characterized by increased infiltration of CD8+ tumor-infiltrating lymphocytes and higher expression of certain immune checkpoint molecules, including CTLA-4, reflecting a more active host immune response. However, studies evaluating the relationship between HPV status, PD-L1 expression, and the efficacy of immune checkpoint inhibitor (ICI) therapy have yielded inconsistent results. In the KEYNOTE-012, KEYNOTE-055, and CheckMate-141 trials, responses to immunotherapy were observed in both HPV-positive and HPV-negative patients, although some analyses demonstrated more favorable outcomes in patients with HPV-positive tumors [43,44,45,46,47]. Although the role of HPV in shaping the tumor immune microenvironment is increasingly recognized, further studies are needed to clarify its relationship with immune checkpoint molecule expression and response to immunotherapy.

Another limitation of the study is that PD-L1 levels were assessed using only the Tumor Proportion Score (TPS), omitting its expression on tumor-infiltrating immune cells as evaluated by the Combined Positive Score (CPS). Although CPS is currently preferred for selecting head and neck squamous cell carcinoma patients for pembrolizumab therapy, TPS remains one of the two most commonly used methods for determining PD-L1 positivity. Previous studies have demonstrated that at high levels (TPS ≥ 50% and CPS ≥ 50), the two scoring systems may be used interchangeably to establish PD-L1 status, although CPS is generally considered a more sensitive measure [23,48]. In our study, the use of TPS was consistent with the study design, as all investigated biomarkers (PD-L1, CTLA-4, IL-10, and IL-18) were evaluated exclusively in tumor cells, ensuring methodological consistency and allowing direct comparison among the analyzed biomarkers. Nevertheless, incorporating CPS assessment alongside TPS could further enhance the clinical relevance of the findings.

## 5. Conclusions

The present study demonstrated a strong association between PD-L1 and CTLA-4 expression. To the best of our knowledge, an association of this magnitude has not previously been reported in OSCC. These findings suggest that both immune checkpoint molecules may contribute to a common immunosuppressive profile within the OSCC tumor microenvironment. The inverse associations observed between IL-18 and both PD-L1 and CTLA-4 expression, together with the positive association between IL-18 and IL-10 expression, highlight the multifaceted nature of immune response regulation. The results further suggest that cytokine-mediated immunosuppressive mechanisms and immune checkpoint pathways may, at least in part, operate independently within the tumor microenvironment. Our study provides novel insights into the co-occurrence of cytokine and immune checkpoint molecule expression in OSCC. Furthermore, it provides a biological rationale for future studies investigating therapeutic strategies targeting multiple immunoregulatory pathways.

## Figures and Tables

**Table 1:** Antibodies used for the assessment of biomarker expression.

Biomarker	Antibody Clone	Manufacturer	Antigen Retrieval	Dilution
IL10	E-10 (mouse monoclonal)	Santa Cruz; Cat. No. sc-8438	TRS pH 9.0	1:400
IL18	IL-18 (rabbit polyclonal)	Abcam; ab191152	TRS pH 6.0	1:300
CTLA-4	CAL49 (rabbit monoclonal)	Abcam; Cat. No. ab237712)	TRS pH 9.0	1:500
PD-L1	22C3 (mouse monoclonal)	Agilent Technologies Cat. No. SK006	TRS pH 6.0	RTU

**Table 2:** Characteristics of the study cohort (*n* = 79).

Variable	Value
Sex	
Female	26 (32.9%)
Male	53 (67.1%)
Age (years)	61.5 ± 10.0
BMI	24.66 ± 4.66
Tumor location	
Tongue	25 (31.6%)
Floor of oral cavity	17 (21.5%)
Gingiva	13 (16.5%)
Buccal mucosa (cheek)	12 (15.2%)
Tongue and floor of oral cavity	6 (7.6%)
Palate	4 (5.1%)
Others	2 (2.5%)
Clinical stage (AJCC)	
I	16 (20.5%)
II	19 (24.4%)
III	18 (23.1%)
IV	25 (32.1%)
Histological grade (G)	
G1	3 (3.8%)
G2	72 (91.1%)
G3	4 (5.1%)
T category (AJCC)	
T1	18 (23.1%)
T2	23 (29.5%)
T3	19 (24.4%)
T4a	18 (23.1%)
N category (AJCC)	
N0	51 (69.9%)
N1	9 (12.3%)
N2	2 (2.7%)
N2a	1 (1.4%)
N2b	7 (9.6%)
N2c	3 (4.1%)
Smoking status	
Yes	39 (49.4%)
No	40 (50.6%)
Alcohol consumption	
Yes	12 (15.2%)
No	67 (84.8%)
Local recurrence	
Yes	25 (31.6%)
No	54 (68.4%)
Nodal recurrence	
Yes	4 (5.1%)
No	75 (94.9%)
Distant metastases	
Yes	7 (8.9%)
No	72 (91.1%)
5-year survival	
Yes	42 (53.2%)
Still alive < 5 years	14 (17.7%)
No	23 (29.1%)

**Table 3:** Expression of CTLA-4, PD-L1, IL-18, IL-10 in the study cohort (*n* = 79).

Variable	Value
CTLA-4 expression	
Less than 1% (negative staining) = 0	10 (12.7%)
1–49% of cells (intermediate expression) = 1	69 (87.3%)
50–100% of cells (strong expression) = 2	0
PD-L1 expression	
Less than 1% (negative staining) = 0	7 (8.9%)
1–49% of cells (intermediate expression) = 1	64 (81.0%)
50–100% of cells (strong expression) = 2	8 (10.1%)
PD-L1 expression (% positive cells), mean ± SD	27.03 ± 17.48
IL-18 expression	
≤10% positive cells = 0	3 (3.8%)
11–25% positive cells = 1	62 (78.5%)
26–50% positive cells = 2	14 (17.7%)
>50–100% positive cells = 3	0
IL-10 expression	
≤10% positive cells = 0	4 (5.1%)
11–25% positive cells = 1	46 (58.2%)
26–50% positive cells = 2	29 (36.7%)
>50–100% positive cells = 3	0

**Table 4:** PD-L1 and CTLA-4 expression. Table 4 presents the relationship between PD-L1 and CTLA-4 expression in patients with OSCC. A statistically significant and very strong correlation was demonstrated between the analyzed biomarkers (*p* < 0.001; Cramér’s V = 0.823).

CTLA-4	PD-L1						Total	
	Less than 1%		1–49%		50–100%			
	N	%	N	%	N	%	N	%
Less than 1%	7	100.0%	2	3.1%	1	12.5%	10	12.7%
1–49%	0	0.0%	62	96.9%	7	87.5%	69	87.3%
Total	7	100.0%	64	100.0%	8	100.0%	79	100.0%

Test Chi-2: Chi-2 = 53,561, *p* < 0.001, V Cramera = 0.823.

**Table 5:** IL-18 and CTLA-4. Table 5 presents the relationship between IL-18 and CTLA-4 expression in patients with OSCC. A statistically significant association was demonstrated between the analyzed biomarkers (*p* = 0.004; Cramér’s V = 0.322). Higher IL-18 expression (26–50% group) was more frequently associated with absent CTLA-4 expression, whereas lower IL-18 expression (0–25% group) predominated among patients with intermediate CTLA-4 expression (1–49% group).

CTLA-4	IL-18				Total	
	0–25%		26–50%			
	N	%	N	%	N	%
Less than 1%	5	7.7%	5	35.7%	10	12.7%
1–49%	60	92.3%	9	64.3%	69	87.3%
Total	65	100.0%	14	100.0%	79	100.0%

Test Chi-2: Chi-2 = 8181, *p* = 0.004, V Cramera = 0.322.

**Table 6:** IL-18 and PD-L1. Table 6 presents the relationship between IL-18 and PD-L1 expression in patients with OSCC (*p* = 0.017; Cramér’s V = 0.322). Higher IL-18 expression (26–50% group) was more frequently associated with absent PD-L1 expression, whereas intermediate PD-L1 expression (1–49%) predominated among patients with lower IL-18 levels (0–25% group).

PD-L1	IL-18				Total	
	0–25%		26–50%			
	N	%	N	%	N	%
Less than 1%	3	4.6%	4	28.6%	7	8.9%
1–49%	55	84.6%	9	64.3%	64	81.0%
50–100%	7	10.8%	1	7.1%	8	10.1%
Total	65	100.0%	14	100.0%	79	100.0%

Test Chi-2: Chi-2 = 8198, *p* = 0.017, V Cramera = 0.322.

**Table 7:** (a) IL-10 and CTLA-4. (b) IL-10 and PD-L1. Table 7a,b present the relationships between IL-10 expression and the expression of CTLA-4 and PD-L1 in patients with OSCC. No statistically significant associations were observed between IL-10 and CTLA-4 expression (*p* = 0.817) or between IL-10 and PD-L1 expression (*p* = 0.319). Intermediate PD-L1 expression (1–49%) and CTLA-4 expression (1–49%) predominated regardless of IL-10 expression level.

**(a)**						
**CTLA-4**	**IL-10**				**Total**	
	**0–25%**		**26–50%**			
	**N**	**%**	**N**	**%**	**N**	**%**
Less than 1%	6	12.0%	4	13.8%	10	12.7%
1–49%	44	88.0%	25	86.2%	69	87.3%
Total	50	100.0%	29	100.0%	79	100.0%
**(b)**						
**PD-L1**	**IL-10**				**Total**	
	**0–25%**		**26–50%**			
	**N**	**%**	**N**	**%**	**N**	**%**
Less than 1%	4	8.0%	3	10.3%	7	8.9%
1–49%	39	78.0%	25	86.2%	64	81.0%
50–100%	7	14.0%	1	3.4%	8	10.1%
Total	50	100.0%	29	100.0%	79	100.0%

(a) Test Chi-2: Chi-2 = 0.053, *p* = 0.817. (b) Test Chi-2: Chi-2 = 2.285, *p* = 0.319.

**Table 8:** IL-10 and IL-18. Table 8 presents the relationship between IL-18 and IL-10 expression in patients with OSCC (*p* < 0.001; Cramér’s V = 0.403). Higher IL-18 expression was more frequently associated with elevated IL-10 expression, whereas lower IL-10 levels predominated among patients with lower IL-18 expression.

IL-18	IL-10				Total	
	0–25%		26–50%			
	N	%	N	%	N	%
0–25%	47	94.0%	18	62.1%	65	82.3%
26–50%	3	6.0%	11	37.9%	14	17.7%
Total	50	100.0%	29	100.0%	79	100.0%

Test Chi-2: Chi-2 = 12,835, *p* < 0.001, V Cramera = 0.403.

**Table 9:** IL-10 and 5-year survival. Table 9 presents the relationship between IL-10 expression and 5-year survival in patients with OSCC (*p* = 0.009; Cramér’s V = 0.290). Higher IL-10 expression was more frequently associated with failure to achieve 5-year survival, whereas lower IL-10 expression predominated among 5-year survivors.

5-Year Survival	IL-10				Total	
	0–25%		26–50%			
	N	%	N	%	N	%
yes	29	58.0%	13	44.8%	42	53.2%
Still alive < 5 year	12	24.0%	2	6.9%	14	17.7%
No	9	18.0%	14	48.3%	23	29.1%
Total	50	100.0%	29	100.0%	79	100.0%

Test Chi-2: Chi-2 = 9408, *p* = 0.009, V Cramera = 0.290.

**Table 10:** Multivariate logistic regression analysis for 5-year survival in patients with OSCC. Table 10 presents the results of multivariate logistic regression analysis for 5-year survival in patients with OSCC. Tumor stage remained the only independent prognostic factor significantly associated with 5-year survival (*p* = 0.002). IL-10 expression and age did not retain statistical significance in the multivariate model.

Variable	OR	95% CI	*p*-Value
IL-10 expression	0.76	0.03–10.11	0.835
Age	0.95	0.89–1.01	0.115
Early stage (I–II vs. III–IV)	7.53	2.25–30.38	0.002 *

OR-odds ratio, CI-confidence interval. Early stage included stage I–II tumors, advanced stage included stage III–IV tumors. *-Statistically significant (p < 0.05).

## Data Availability

The raw data supporting the findings of this study are available from the corresponding author upon reasonable request.

## Supplementary Materials

The following supporting information can be downloaded at: https://www.mdpi.com/article/10.3390/cimb48080785/s1.
