# Supplementary material for: Interrelationships Between PD-L1, CTLA-4, IL-10 and IL-18 Expression in Oral Squamous Cell Carcinoma
Source: Curr Issues Mol Biol. 2026 Aug 1;48(8):785. doi: 10.3390/cimb48080785 (PMC13511622; doi:10.3390/cimb48080785)
Supplement: Supplementary file 1 [file cimb-48-00785-s001.zip › cimb-4402045-supplementary.pdf]

Table S1. Associations between CTLA-4 expression and selected clinicopathological factors (sex, tumor location, clinical stage, T category, N category, smoking, alcohol use, 5-years survival, distant metastases, local recurrence).

**Associations between CTLA-4 expression and selected clinicopathological factors**					
**Variable**	**Category**	**CTLA-4 less than 1% n (%)**	**CTLA-4 1-49% n (%)**	**Total n**	**p-value**
Sex	Female	3 (11.5%)	23 (88.5%)	26	0.834
	Male	7 (13.2%)	46 (86.8%)	53	
Tumor location	Tongue	3 (12.0%)	22 (88.0%)	25	0.256
	Floor of oral cavity	5 (29.4%)	12 (70.6%)	17	
	Gingiva	1 (7.7%)	12 (92.3%)	13	
	Cheek	1 (8.3%)	11 (91.7%)	12	
	Others	0 (0.0%)	6 (100.0%)	6	
	Tongue and floor	0 (0.0%)	6 (100.0%)	6	
Clinical stage	I	2 (12.5%)	14 (87.5%)	16	0.372
	II	4 (21.1%)	15 (78.9%)	19	
	III	3 (16.7%)	15 (83.3%)	18	
	IV	1 (4.0%)	24 (96.0%)	25	
T category	T1	2 (11.1%)	16 (88.9%)	18	0.233
	T2	4 (17.4%)	19 (82.6%)	23	
	T3	4 (21.1%)	15 (78.9%)	19	
	T4a	0 (0.0%)	18 (100.0%)	18	
N category	N0	8 (15.7%)	43 (84.3%)	51	0.358
	N1	0 (0.0%)	9 (100.0%)	9	
	N2 (A,B,C)	1 (7.7%)	12 (92.3%)	13	
Smoking	Yes	6 (15.4%)	33 (84.6%)	39	0.472
	No	4 (10.0%)	36 (90.0%)	40	
Alcohol use	Yes	2 (16.7%)	10 (83.3%)	12	0.650
	No	8 (11.9%)	59 (88.1%)	67	
5-year survival	Yes	5 (50.0%)	37 (53.6%)	42	0.516
	Still alive <5 years	3 (30.0%)	11 (15.9%)	14	
	No	2 (20.0%)	21 (30.4%)	23	
Distant metastases	Yes	0 (0.0%)	7 (10.1%)	7	0.291
	No	10 (100.0%)	62 (89.9%)	72	
Local recurrence	Yes	2 (20.0%)	23 (33.3%)	25	0.397
	No	8 (80.0%)	46 (66.7%)	54	

No statistically significant associations were observed between CTLA-4 expression and the analyzed clinicopathological parameters, including sex, tumor location, clinical stage, T and N category, smoking status, alcohol consumption, distant metastases, local recurrence, or 5-year survival ($p > 0.05$).

Table S2. Associations between PD-L1 expression and selected clinicopathological factors (sex, tumor location, clinical stage, T category, N category, smoking, alcohol use, 5-years survival, distant metastases, local recurrence), regression model and ROC analysis.

**Associations between PD-L1 expression and selected clinicopathological factors**						
**Variable**	**Category**	**No expression n (%)**	**1–49% n (%)**	**50–100% n (%)**	**Total n**	**p-value**
Sex	Female	3 (11.5%)	19 (73.1%)	4 (15.4%)	26	0.433
	Male	4 (7.5%)	45 (84.9%)	4 (7.5%)	53	
Tumor localization	Tongue	3 (12.0%)	20 (80.0%)	2 (8.0%)	25	0.947
	Floor of oral cavity	2 (11.8%)	13 (76.5%)	2 (11.8%)	17	
	Gingiva	1 (7.7%)	10 (76.9%)	2 (15.4%)	13	
	Cheek	1 (8.3%)	11 (91.7%)	0 (0.0%)	12	
	Tongue and floor of oral cavity	0 (0.0%)	5 (83.3%)	1 (16.7%)	6	
	Others	0 (0.0%)	5 (83.3%)	1 (16.7%)	6	
Clinical stage	I	1 (6.3%)	14 (87.5%)	1 (6.3%)	16	0.869
	II	3 (15.8%)	14 (73.7%)	2 (10.5%)	19	
	III	2 (11.1%)	14 (77.8%)	2 (11.1%)	18	
	IV	1 (4.0%)	21 (84.0%)	3 (12.0%)	25	
T category	T1	1 (5.6%)	15 (83.3%)	2 (11.1%)	18	0.523
	T2	3 (13.0%)	19 (82.6%)	1 (4.3%)	23	
	T3	3 (15.8%)	13 (68.4%)	3 (15.8%)	19	
	T4a	0 (0.0%)	16 (88.9%)	2 (11.1%)	18	
N category	N0	5 (9.8%)	42 (82.4%)	4 (7.8%)	51	0.803
	N1	0 (0.0%)	8 (88.9%)	1 (11.1%)	9	
	N2 (A,B,C)	1 (7.7%)	10 (76.9%)	2 (15.4%)	13	
Smoking	Yes	4 (10.3%)	31 (79.5%)	4 (10.3%)	39	0.908
	No	3 (7.5%)	33 (82.5%)	4 (10.0%)	40	
Alcohol use	Yes	1 (8.3%)	10 (83.3%)	1 (8.3%)	12	0.971
	No	6 (9.0%)	54 (80.6%)	7 (10.4%)	67	
5-year survival	Yes	3 (42.9%)	34 (53.1%)	5 (62.5%)	42	0.921
	Still alive <5 years	2 (28.6%)	11 (17.2%)	1 (12.5%)	14	
	No	2 (28.6%)	19 (29.7%)	2 (25.0%)	23	
Distant metastases	Yes	0 (0.0%)	7 (10.9%)	0 (0.0%)	7	0.407
	No	7 (100.0%)	57 (89.1%)	8 (100.0%)	72	
Local recurrence	Yes	1 (14.3%)	21 (32.8%)	3 (37.5%)	25	0.565
	No	6 (85.7%)	43 (67.2%)	5 (62.5%)	54	
Regression model	Age	-	Beta = -0.131	-	-	0.534
	BMI	-	Beta = -0.067	-	-	0.882
ROC analysis	PD-L1	AUC = 0.557	Cut-off = 35	OMC = 0.41	-	0.430

No statistically significant associations were observed between PD-L1 expression and the analyzed clinicopathological parameters, including sex, tumor location, clinical stage, T and N category, smoking status, alcohol consumption, distant metastases, local recurrence, or 5-year survival ($p > 0.05$). Additionally, age and BMI were not significantly associated with PD-L1 expression.

Table S3. Associations between IL-18 expression and selected clinicopathological factors (sex, tumor location, clinical stage, T category, N category, smoking, alcohol use, 5-years survival, distant metastases, local recurrence).

**Associations between IL-18 expression and selected clinicopathological factors**					
**Variable**	**Category**	**IL-18 0–25% n (%)**	**IL-18 26-50% n (%)**	**Total n**	**p-value**
Sex	Female	22 (84.6%)	4 (15.4%)	26	0.703
	Male	43 (81.1%)	10 (18.9%)	53	
Tumor localization	Tongue	21 (84.0%)	4 (16.0%)	25	0.997
	Floor of oral cavity	14 (82.4%)	3 (17.6%)	17	
	Gingiva	10 (76.9%)	3 (23.1%)	13	
	Cheek	10 (83.3%)	2 (16.7%)	12	
	Tongue and floor	5 (83.3%)	1 (16.7%)	6	
	Others	5 (83.3%)	1 (16.7%)	6	
Clinical stage	I	14 (87.5%)	2 (12.5%)	16	0.740
	II	15 (78.9%)	4 (21.1%)	19	
	III	14 (77.8%)	4 (22.2%)	18	
	IV	22 (88.0%)	3 (12.0%)	25	
T category	T1	16 (88.9%)	2 (11.1%)	18	0.685
	T2	18 (78.3%)	5 (21.7%)	23	
	T3	15 (78.9%)	4 (21.1%)	19	
	T4a	16 (88.9%)	2 (11.1%)	18	
N category	N0	44 (86.3%)	7 (13.7%)	51	0.341
	N1	6 (66.7%)	3 (33.3%)	9	
	N2 (A,B,C)	11 (84.6%)	2 (15.4%)	13	
Smoking	Yes	31 (79.5%)	8 (20.5%)	39	0.521
	No	34 (85.0%)	6 (15.0%)	40	
Alcohol use	Yes	10 (83.3%)	2 (16.7%)	12	0.917
	No	55 (82.1%)	12 (17.9%)	67	
5-year survival	Yes	35 (53.8%)	7 (50.0%)	42	0.331
	Still alive <5 years	13 (20.0%)	1 (7.1%)	14	
	No	17 (26.2%)	6 (42.9%)	23	
Distant metastases	Yes	6 (9.2%)	1 (7.1%)	7	0.803
	No	59 (90.8%)	13 (92.9%)	72	
Local recurrence	Yes	18 (27.7%)	7 (50.0%)	25	0.104
	No	47 (72.3%)	7 (50.0%)	54	

No statistically significant associations were observed between IL-18 expression and the analyzed clinicopathological parameters, including sex, tumor location, clinical stage, T and N category, smoking status, alcohol consumption, distant metastases, local recurrence, or 5-year survival ($p > 0.05$).

Table S4. Associations between IL-10 expression and selected clinicopathological factors (sex, tumor location, clinical stage, T category, N category, smoking, alcohol use, 5-years survival, distant metastases, local recurrence).

Associations between IL-10 expression and selected clinicopathological factors					
**Variable**	**Category**	**IL-10 0–25% n (%)**	**IL-10 26-50% n (%)**	**Total n**	**p-value**
Sex	Female	17 (65.4%)	9 (34.6%)	26	0.738
	Male	33 (62.3%)	20 (37.7%)	53	
Tumor localization	Tongue	18 (72.0%)	7 (28.0%)	25	0.492
	Floor of oral cavity	13 (76.5%)	4 (23.5%)	17	
	Gingiva	7 (53.8%)	6 (46.2%)	13	
	Cheek	6 (50.0%)	6 (50.0%)	12	
	Tongue and floor	3 (50.0%)	3 (50.0%)	6	
	Others	3 (50.0%)	3 (50.0%)	6	
Clinical stage	I	11 (68.8%)	5 (31.3%)	16	0.924
	II	11 (57.9%)	8 (42.1%)	19	
	III	11 (61.1%)	7 (38.9%)	18	
	IV	16 (64.0%)	9 (36.0%)	25	
T category	T1	13 (72.2%)	5 (27.8%)	18	0.778
	T2	13 (56.5%)	10 (43.5%)	23	
	T3	12 (63.2%)	7 (36.8%)	19	
	T4a	11 (61.1%)	7 (38.9%)	18	
N category	N0	32 (62.7%)	19 (37.3%)	51	0.889
	N1	6 (66.7%)	3 (33.3%)	9	
	N2 (A,B,C)	9 (69.2%)	4 (30.8%)	13	
Smoking	Yes	27 (69.2%)	12 (30.8%)	39	0.279
	No	23 (57.5%)	17 (42.5%)	40	
Alcohol use	Yes	9 (75.0%)	3 (25.0%)	12	0.361
	No	41 (61.2%)	26 (38.8%)	67	
Distant metastases	Yes	5 (10.0%)	2 (6.9%)	7	0.640
	No	45 (90.0%)	27 (93.1%)	72	
Local recurrence	Yes	16 (32.0%)	9 (31.0%)	25	0.929
	No	34 (68.0%)	20 (69.0%)	54	
5-year survival	Yes	29 (58.0%)	13 (44.8%)	42	0.009
	Still alive <5 years	12 (24.0%)	2 (6.9%)	14	
	No	9 (18.0%)	14 (48.3%)	23	

IL-10 expression showed no significant associations with most analyzed clinicopathological parameters, including sex, tumor location, clinical stage, T and N category, smoking status, alcohol consumption, distant metastases, or local recurrence ($p > 0.05$). Higher IL-10 expression was significantly associated with poorer 5-year survival in univariate analysis ($p<0.009$).

Table S5. Comparison of patients with different biomarkers expression levels in terms of age and BMI.

Comparison of patients with different biomarker expression levels in terms of age and BMI				
**Biomarker**	**Variable**	**Lower expression**	**Higher expression**	**p-value**
CTLA-4	BMI	24.77 ± 5.37	24.64 ± 4.59	0.140
CTLA-4	Age	65.60 ± 5.99	60.94 ± 10.35	0.947
PD-L1	BMI	23.04 ± 4.14 / 24.81 ± 4.90 / 24.84 ± 2.91	-	0.936
PD-L1	Age	66.29 ± 6.75 / 61.00 ± 10.69 / 61.63 ± 4.50	-	0.943
IL-18	BMI	24.41 ± 4.42	25.81 ± 5.71	0.488
IL-18	Age	61.17 ± 10.01	63.21 ± 10.14	0.225
IL-10	BMI	24.19 ± 5.02	25.46 ± 3.92	0.198
IL-10	Age	60.28 ± 9.37	63.69 ± 10.82	0.304

No statistically significant differences in age or BMI were observed between patients with different expression levels of CTLA-4, PD-L1, IL-18, or IL-10 ($p > 0.05$).
